# Association between viral seasonality and meteorological factors

**DOI:** 10.1038/s41598-018-37481-y

**Published:** 2019-01-30

**Authors:** Rory Henry Macgregor Price, Catriona Graham, Sandeep Ramalingam

**Affiliations:** 1 Department of Laboratory Medicine, Royal Infirmary of Edinburgh, Edinburgh, UK; 2 Division of Infection and Pathway Medicine, Edinburgh Medical School, Division of Infection and Pathway Medicine, University of Edinburgh, Edinburgh, UK; 3 Wellcome Trust Clinical Research Facility, University of Edinburgh, Edinburgh, UK

## Abstract

Numerous viruses can cause upper respiratory tract infections. They often precede serious lower respiratory tract infections. Each virus has a seasonal pattern, with peaks in activity in different seasons. We examined the effects of daily local meteorological data (temperature, relative humidity, “humidity-range” and dew point) from Edinburgh, Scotland on the seasonal variations in viral transmission. We identified the seasonality of rhinovirus, adenovirus, influenza A and B viruses, human parainfluenza viruses 1–3 (HPIV), respiratory syncytial virus (RSV) and human metapneumovirus (HMPV) from the 52060 respiratory samples tested between 2009 and 2015 and then confirmed the same by a generalised linear model. We also investigated the relationship between meteorological factors and viral seasonality. Non-enveloped viruses were present throughout the year. Following logistic regression adenovirus, influenza viruses A, B, RSV and HMPV preferred low temperatures; RSV and influenza A virus preferred a narrow “humidity-range” and HPIV type 3 preferred the season with lower humidity. A change (i.e. increase or decrease) in specific meteorological factors is associated with an increase in activity of specific viruses at certain times of the year.

## Introduction

The common cold is typically a mild upper respiratory tract infection (URTI), with symptoms such as nasal stuffiness and discharge, sore throat, coughing and sneezing^1,2^. URTI are mostly self-limiting but can progress to lower respiratory tract infections (LRTI) particularly in those with underlying conditions^3,4^. Adults suffer from a cold two to five times a year, and children can have 7–10 episodes annually^5^. Numerous viruses can cause URTI^6^. Rhinoviruses (also called the common cold virus), are responsible for around 30% to 70% of all respiratory infections^5,6^. Since there are >100 different rhinovirus types, reinfections are very common^7^. Coronavirus is the second most common cause of URTI, causing 7% to 18% of cases^8^. Other viruses including respiratory syncytial viruses (RSV) types A and B, human parainfluenza viruses (HPIV) types 1–4, adenoviruses, human metapneumovirus (HMPV) and influenza viruses A-C (IAV, IBV and ICV) also cause URTI^8,9^.

Most respiratory viral infections have seasonality. In temperate regions, URTI increase in frequency in autumn and spring, and remain raised through winter^8^. Three theories have been put forward to explain viral seasonality. 1: The effect of climatic conditions on host resistance to infection (low vitamin D levels following lack of sun exposure can affect our ability to fight infection)^9,10^. 2: The effect of meteorological factors (e.g. temperature, humidity) on virus survival and hence on infection rates^9,11^. 3: The effect of behavioural changes on transmission (e.g. spending more time indoors in close proximity to others or aggregation of susceptible children at schools during the colder months)^8,9^. There are other hypothesis such as diminished immune responses in a chilled host, or the reactivation of dormant viruses by chilling for the increase in URTI during the colder months^12^.

Temperature has an important effect on viral activity, particularly in the case of enveloped viruses. In a guinea pig model, IAV transmission is more effective in cold and dry conditions^13^. Integrity of IAV envelopes are better at lower temperatures, whilst in warm temperatures, the envelope becomes disordered and the virus susceptible to damage^14^. Reports suggest that IAV survival is better associated with absolute humidity (which measures the amount of water vapour in air regardless of the temperature) rather than relative humidity^15,16^. An association of absolute humidity with hospitalisation due to influenza LRTI (but not with LRTI due to Rhinovirus or RSV) has recently been reported^17^.

Most reports on viral seasonality concentrate on viruses that appear in the winter months, particularly influenza viruses and RSV. Here we aim to determine the seasonality of all the respiratory viruses detected over 6.5 years, and identify any associations between changes in meteorological factors (such as temperature or humidity) and the appearance of different viruses across a whole year.

## Results

Between April 2009 and November 2015, rhinoviruses and adenoviruses were present throughout the year. Rhinoviruses were present most days of the year (84.7%), followed by adenovirus which was present on 52.3% of the days. RSV (36.4%), HMPV (32.8%) and IAV (32.7%) were present in around 1/3^rd^ of the year. HPIV-3 was present in 24.6% of days, followed by HPIV-1 (15.5%) and IBV (15.2%). HPIV-2 was present for the fewest number of days (6.5%) (Table 1). Children ≤10 accounted for 45% of the number of samples tested. The age groups of those tested are in Table 2.

**Table 1:** Results of respiratory samples for each virus.

Virus	No. Positive Samples	No. Negative Samples	Total No.Samples	Days Positive	No. days tested for
Rhinovirus	5881	42893	48774	84.7%	1949
Adenovirus	2005	48730	50735	52.3%	2148
RSV	1530	50527	52057	36.4%	2168
IAV	2225	49835	52060	32.7%	2168
IBV	656	50610	51266	15.2%	2168
HPIV-1	410	48040	48450	15.5%	2142
HPIV-2	137	48314	48451	6.5%	2142
HPIV-3	893	47556	48449	24.6%	2142
HMPV	929	48018	48947	32.8%	1959

**Table 2:** Age demographics of the patient cohort.

Age group	<1	1–10	11–20	21–30	31–40	41–50	51–60	61–70	71–80	81–90	>90
Number of patients	4270	11939	2152	1901	1910	2399	2609	3165	3092	2277	477
%	12%	33%	6%	5%	5%	7%	7%	9%	9%	6%	1%

**Figure 1:**
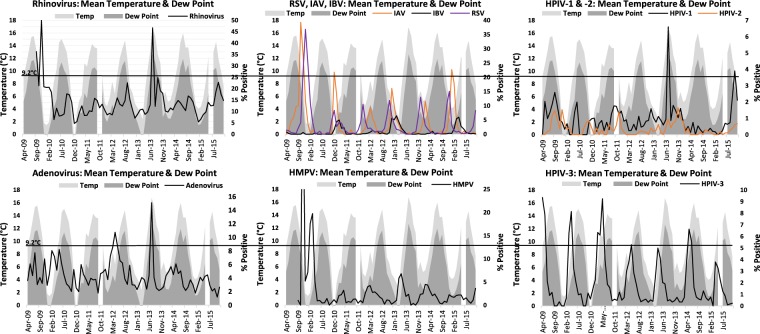
Comparison of viral seasonality, mean temperature and mean dew point. X axis: Month/Year Y axis: Monthly mean temperature and dew points (shaded area) over the study period and % positivity for each virus (coloured lines, scale differs between viruses). Horizontal line is the average temperature (9.2 °C) across the study period. The line representing the % positivity for each virus demonstrates viral seasonality through the year. It can be compared to the changes in temperature and dew point through the year. In November 2009, HMPV tests were positive 50% of the time, and hence that month’s results are high in comparison to all other results. The high prevalence of rhinovirus and HMPV in 2009 may reflect selective testing for these viruses when the test was introduced.

**Figure 2:**
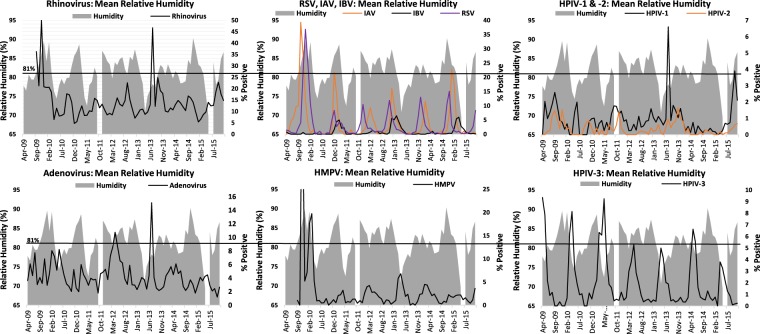
Comparison of viral seasonality and mean relative humidity. X axis: Month/Year. Y axis: Monthly mean relative humidity (shaded area) over the study period and % positivity for each virus (coloured lines, scale differs between viruses). Horizontal line is the average relative humidity (81%) across the study period. The line representing the % positivity for each virus demonstrates viral seasonality through the year. It can be compared to the changes in mean relative humidity through the year. In November 2009, HMPV tests were positive 50% of the time, and hence that month’s results are high in comparison to all other results. The high prevalence of rhinovirus and HMPV in 2009 may reflect selective testing for these viruses when the test was introduced.

**Figure 3:**
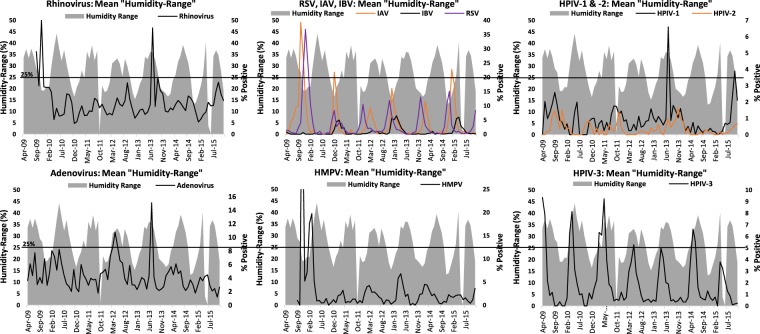
Comparison of viral seasonality and humidity-range. X axis: Month/Year. Y axis: Monthly mean “humidity-range” (shaded area) over the study period and % positivity for each virus (coloured lines, scale differs between viruses). Horizontal line is the average “humidity-range” (25%) across the study period. The line representing the % positivity for each virus demonstrates viral seasonality through the year. It can be compared to the changes in “humidity-range” through the year. In November 2009, HMPV tests were positive 50% of the time, and hence that month’s results are high in comparison to all other results. The high prevalence of rhinovirus and HMPV in 2009 may reflect selective testing for these viruses when the test was introduced.

Figures 1–3 plot the weather conditions and the virus prevalence during the study period. Mean air temperature (Fig. 1) showed the expected seasonality, with a peak in July (14 °C to 16 °C) and a trough in January (−1 °C to 4 °C). Mean dew point (Fig. 1) and the variation in humidity within a day (i.e. “humidity-range”, Fig. 3) showed a similar pattern. Mean dew point was highest in August (10 °C to 12 °C) and lowest in February (−2 °C to 3 °C). “Humidity-range” was larger in the summer months (40% to 44%), and least variable in winter (15% to 18%). Mean relative humidity however showed the opposite pattern, typically highest in December (88% to 91%) and lowest in June (70% to 74%). Air pressure, wind speed and duration of daylight did not show clear seasonality (data not shown).

Many viruses have a definite seasonality (Figs 1–3). RSV and IAV have the largest seasonal peaks, appearing in November-December and December-January respectively. IBV (February-March), HMPV (March), and HPIV-3 (April-May) follow sequentially. IBV has a biennial distribution occurring every other year. HPIV-2 has two peaks, a major peak around October-November and a minor peak in July. A clear seasonality is not noticeable for HPIV-1. Adenovirus and rhinovirus are present throughout the year. Rhinovirus also has two peaks, a major peak around October-November and a minor peak in March. Adenovirus has a smaller peak in March-April.

Temperature appears to have the greatest effect on viral seasonality (Fig. 1). The average temperature during the study was 9.2 °C. In keeping with their year-round presence, the mean temperature when adenovirus and rhinovirus are present is closer to the annual mean at 8.8 °C and 8.9 °C respectively. A preference for colder temperatures is noticeable for RSV, IAV, and IBV (6.3 °C, 6.7 °C and 6.3 °C respectively). HMPV is most prevalent when the temperature becomes slightly warmer (7.4 °C). HPIV-3 is the only virus to prefer above average temperatures (9.4 °C). The average dew point was 6.0 °C (Fig. 1). Dew point has a similar pattern of relationship to viruses as temperature, with RSV (3.7 °C), IAV (3.9 °C), IBV (3.3 °C) and HMPV (4.4 °C) preferring lower dew points. In contrast, HPIV-3 prefers a higher dew point (5.9 °C). Temperature and dew point are both significantly lower on days adenovirus, RSV, IAV, IBV, and HMPV are present (Table 3).

**Table 3:** Comparison of mean meteorological values on the days a virus is detectable or undetectable.

Agent/(Number of days tested)	Meteorological factors	Mean on days virus was		Difference in means	95% CI		p Value*
		Detected	Not detected		Lower	Upper	
Adenoviruses (2148)	Temperature (°C)	8.83	9.44	−0.61	−1.021	−0.199	**0**.**004**
	Dew Point (°C)	5.70	6.28	−0.58	−0.971	−0.192	**0**.**003**
	Relative Humidity (%)	81.67	81.53	0.15	−0.545	0.843	0.674
	Humidity-range (%)	26.76	27.83	−1.07	−2.15	0.01	0.052
Rhinoviruses (1949)	Temperature (°C)	8.92	8.64	0.28	−0.324	0.883	0.365
	Dew Point (°C)	5.78	5.55	0.23	−0.373	0.833	0.454
	Relative Humidity (%)	81.56	81.86	−0.29	−1.291	0.706	0.565
	Humidity-range (%)	26.91	27.30	−0.39	−2.012	1.239	0.640
RSV (2168)	Temperature (°C)	6.27	10.69	−4.42	−4.801	−4.035	**<0**.**001**
	Dew Point (°C)	3.70	7.24	−3.55	−3.926	−3.172	<**0**.**001**
	Relative Humidity	83.96	80.30	3.67	2.964	4.365	<**0**.**001**
	Humidity-range (%)	21.79	30.40	−8.61	−9.672	−7.557	<**0**.**001**
IAV (2168)	Temperature (°C)	6.73	10.22	−3.50	−3.912	−3.079	**<0**.**001**
	(%)Dew Point (°C)	3.94	6.93	−2.99	−3.384	−2.586	<**0**.**001**
	Relative Humidity	82.93	80.99	1.94	1.203	2.666	<**0**.**001**
	Humidity-range (%)	23.82	28.94	−5.11	−6.238	−3.985	<**0**.**001**
IBV (2168)	Temperature (°C)	6.28	9.67	−3.39	−3.951	−2.828	**<0**.**001**
	Dew Point (°C)	3.26	6.52	3.26	−3.793	−2.726	<**0**.**001**
	Relative Humidity (%)	81.70	81.67	0.03	−0.987	1.049	0.952
	Humidity-range (%)	24.60	27.87	−3.27	−4.805	−1.743	<**0**.**001**
HPIV-1 (2142)	Temperature (°C)	9.03	9.14	−0.11	−0.659	0.442	0.698
	Dew Point (°C)	6.08	5.97	0.11	−0.413	0.634	0.689
	Relative Humidity (%)	82.55	81.42	1.13	0.173	2.089	**0**.**021**
	Humidity-range (%)	25.85	27.56	−1.71	−3.197	−0.216	**0**.**025**
HPIV-2 (2142)	Temperature (°C)	8.22	9.19	−0.96	−1.824	−0.103	0.280
	Dew Point (°C)	5.26	6.03	−0.78	−1.619	0.07	0.72
	Relative Humidity (%)	82.21	81.56	0.65	−0.687	1.995	0.337
	Humidity-range (%)	26.42	27.35	−0.93	−3.036	1.179	0.386
HPIV-3 (2142)	Temperature (°C)	9.36	9.05	0.32	−0.166	−0.796	0.199
	Dew Point (°C)	5.86	6.02	−0.16	−0.617	0.298	0.495
	Relative Humidity (%)	79.86	82.16	−2.30	−3.102	−1.498	<**0**.**001**
	Humidity-range (%)	29.68	26.51	3.18	1.926	4.424	<**0**.**001**
HMPV (1959)	Temperature (°C)	7.42	9.56	−2.15	−2.601	−1.698	**<0**.**001**
	Dew Point (°C)	4.35	6.42	−2.06	−2.493	−1.637	<**0**.**001**
	Relative Humidity (%)	81.75	81.62	0.13	−0.665	0.925	0.748
	Humidity-range (%)	25.81	27.44	−1.63	−2.833	−0.421	**0**.**008**

CI: Confidence interval, p value from T test. Significant p values in bold. We measured the mean of each meteorological factor on days when we received positive samples for a given virus and on days where we did not receive a positive sample for that virus. Hence a larger difference in means denotes that a virus is commonly present at an extreme of that meteorological factor: e.g. RSV more often being present when temperature is lower.

The average relative humidity was 81% over the study period (Fig. 2). RSV (84%), IAV (82.9%) and HPIV-1(82.6%) prefer higher relative humidity. HPIV-3 is more active when the relative humidity is lower (79.9%) (Table 3). The average variation in humidity within the day (i.e. “humidity-range”) was 25%. “Humidity-range” is significantly narrow for most viruses apart from rhinovirus, HPIV-2 and HPIV-3 (Table 3). RSV (21.8%) and IAV (23.8%) prefer much narrower ranges then other viruses. HPIV-3, in contrast to the other viruses, appears to prefer a wide “humidity-range” (29.7%). Though HPIV-2 seems to prefer raised humidity and a narrow “humidity-range”, the difference in these variables between days the HPIV-2 is present or absent is insignificant (Table 3), potentially a reflection of the lower number of HPIV-2 cases.

Table 4 shows the results of the binomial logistic regression carried out to determine the effect of multiple metrological factors on virus activity. It demonstrates how a unit increase in each meteorological variable (either 1 °C for mean temperature, or 1% for mean relative humidity or “humidity-range”) alters the odds of a virus being present. Only results that were found to be significant in the t-test were examined using logistic regression (i.e. rhinovirus and HPIV-2 were excluded). Following the logistic regression, no significant association was found for HPIV-1. A mean temperature increase by 1 °C reduces the odds of detecting adenovirus, RSV, IAV, IBV and HMPV. Adenovirus has a small decrease (2.8%), while RSV (17.3%), IAV (13.7%), IBV (13%) and HMPV (9.9%) have larger decreases. A 1% increase in relative humidity reduces the odds of HPIV-3 being present (2.6%). A 1% increase in “humidity-range” also decreases the odds of detecting RSV (3.8%) and IAV (1.7%). Air temperature has a significant effect against the most number of viruses (n = 5) followed by “humidity-range” and relative humidity. The smaller effect of “humidity-range” in comparison to mean temperature may be due to an interrelationship between mean humidity and “humidity-range”, since logistic regression takes multiple variables into account.

**Table 4:** Binomial logistic regression of each virus against each metrological factor.

Virus	Meteorological Factor	p Value	Odds Ratio	95% CI for Odds Ratio	
				Lower	Upper
Adenoviruses	Temperature (°C)	0.001	0.972	0.955	0.988
RSV	Temperature (°C)	<0.001	0.827	0.808	0.847
	Humidity-range (%)	<0.001	0.962	0.952	0.972
IAV	Temperature (°C)	<0.001	0.863	0.844	0.882
	Humidity-range (%)	<0.001	0.983	0.973	0.992
IBV	Temperature (°C)	<0.001	0.870	0.847	0.894
HPIV-3	Humidity Mean (%)	<0.001	0.974	0.959	0.988
HMPV	Temperature (°C)	<0.001	0.901	0.883	0.920

CI: Confidence interval. Only significant results are shown.

**Figure 4:**
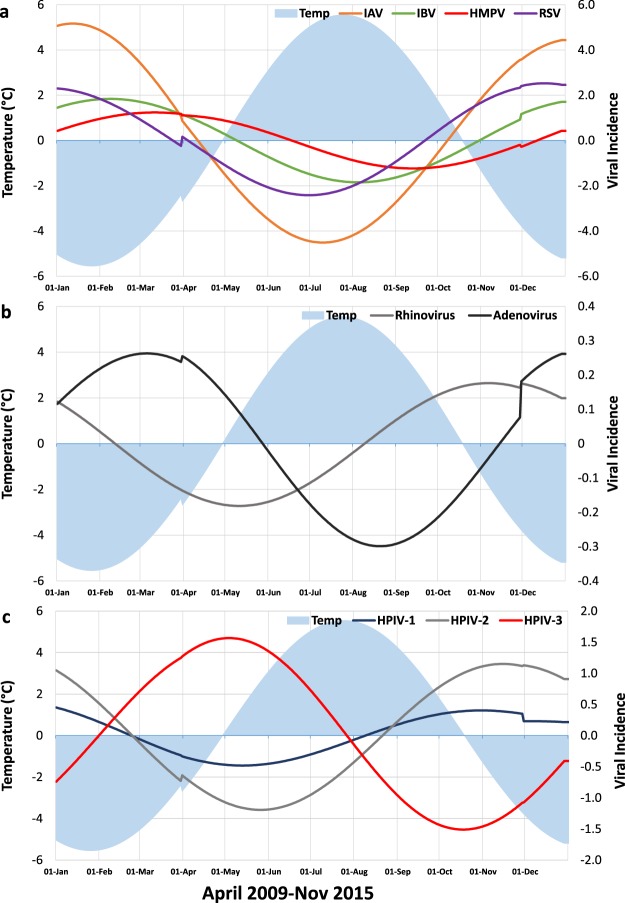
Generalised Linear Models.The generalised linear models were performed to demonstrate the seasonality of each virus. Using temperature as a comparison meteorological factor, the highest point in the waveform for each virus is the time of year where they are most active in the population 4a: Enveloped viruses in the winter. RSV – 17^th^ of December, IAV – 12^th^ of January, IBV – 8^th^ of February; HMPV – 11^th^ of March 4b: Non-enveloped viruses. Adenovirus – 5^th^ of March, Rhinovirus – 6^th^ of November; 4c: Human parainfluenza viruses. HPIV-1–31^st^ of October, HPIV-2–15^th^ of November, HPIV-3–4^th^ of May.

Figure 4a–c are the generalised linear models (GLM), examining each virus’ incidence across a year, averaged over the entire study period. This was compared with temperature over the same period. These models confirm a preference for colder temperatures for RSV, IAV and IBV, and the timings of their seasonal peaks in and around 17^th^ December, 12^th^ January and 8^th^ February respectively. Even though rhinovirus and adenovirus are present throughout the year, their GLM’s point to periods of increased activity (around the 5^th^ of March for Adenovirus and around the 6^th^ November for rhinovirus). The GLM corroborates HPIV-3’s preference for warmer temperatures and its seasonal peak around the 4^th^ of May. Finally, the GLM suggests that HMPV peaks around the 11^th^ of March, HPIV-1 peaks around the 31^st^ October, and HPIV-2 peaks around the 15^th^ of November.

**Figure 5:**
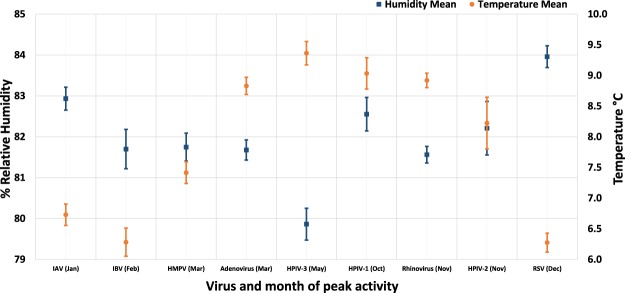
Change in mean temperature and mean relative humidity as individual viruses increase in activity across a year.Results from ANOVA. Changes in mean temperature and mean relative humidity associated with sequential increase in activity of viruses between January and December (vertical lines - standard error of the mean). Each data point corresponds to the mean temperature or mean relative humidity during the period when the virus is most active (e.g. IAV in January).

From Fig. 4a–c, a rough order of seasonal peaks is noticeable for the different viruses, beginning with IAV in January. After this, there are peaks of IBV in February, HMPV and Adenovirus in March, HPIV-3 in May, HPIV-1 in the end of October, rhinoviruses, and HPIV-2 in November, and ending with RSV in December. Since temperature and humidity appear to have a significant impact on viral incidence when analysed individually, we calculated the difference in mean temperature and humidity preferred by the different viruses and arranged them in the order as above (Table 5). Figure 5 represents this pattern visually using results from ANOVA. Starting with the seasonal peak of IAV in January, there is a decrease in relative humidity from 82.9% to 81.7% in February, along with a decrease in mean temperature from 6.7 °C to 6.3 °C. These changes are associated with peaks in incidence of IBV. Following this, relative humidity stabilises until March. However, the mean temperature rises significantly to 7.4 °C in March corresponding with the peak in HMPV and then further to 8.8 °C when a peak in adenovirus occurs. Next, there is a further increase in temperature to 9.4 °C along with a significant reduction in relative humidity to 79.9% in May when a peak in HPIV-3 is observed. A slight drop in mean temperature (8.4 °C) and a significant increase in mean relative humidity (82.6%), from May to October is associated with a peak in HPIV-1. With mean temperatures being fairly constant, a further drop in relative humidity (81.6%) is associated with a peak in rhinovirus activity in November. A slight increase in humidity and a drop in temperature in November is associated with the presence of HPIV-2. A dramatic drop in temperature to 6.2 °C and an increase in relative humidity to 84.0%, leads to the peak in RSV in December. Then the cycle begins anew with IAV in January.

**Table 5:** Comparison of mean temperature and mean humidity for different viruses.

	Temperature/Humidity	IBV	HMPV	Adenovirus	HPIV-3	HPIV-1	Rhinovirus	HPIV-2	RSV
IAV	T	−0.45	0.69	**−2**.10***	**−2**.63***	**−2**.31***	**−2**.19***	**−1**.50*	0.45
	H	−1.23	−1.18	1.26	**3**.07***	0.38	**1**.37**	0.72	−1.03
IBV	T		**1**.13*	**−2**.55***	**−3**.08***	**−2**.75***	**−2**.64***	**−1**.94**	0.01
	H		0.05	0.02	1.84	−0.85	0.14	−0.51	**−2**.26**
HMPV	T			**−1**.42***	**−1**.95***	**−1**.62***	**−1**.51***	−0.81	**1**.14***
	H			0.07	**1**.89**	−0.81	0.19	−0.46	**−2**.21***
Adenovirus	T				−0.53	0.20	−0.09	−0.61	**−2**.56***
	H				**1**.81**	0.88	0.11	0.53	**2**.28***
HPIV-3	T					−0.33	−0.44	−1.14	**−3**.09***
	H					**2**.69***	**1**.70**	2.35	**4**.10***
HPIV-1	T						0.11	−0.81	**−2**.76***
	H						0.99	−0.34	1.41
Rhinovirus	T							−0.70	**−2**.65***
	H							0.65	**2**.40***
HPIV-2	T								**−1**.95***
	H								1.75

To determine which variable significantly affects transition from one virus to another, we calculated the differences in mean temperature and relative humidity of virus in column minus virus in row. e.g. a decrease in temperature from autumn to winter is associated with a change from HPIV-2 to RSV. T = Temperature Difference, H = Humidity difference. Results of ANOVA. Significant p values in bold (*p < 0.05; **p < 0.01; ***p < 0.001).

## Discussion

Du Prel *et al*. have reported that air temperature and relative humidity were important factors affecting viral seasonality in Germany over a 6-year period^18^. As in our study, air pressure did not have a significant correlation. However, to our knowledge, this is the first study to report the association between meteorological conditions and the sequential appearance of viruses through the year and the effect of daily fluctuations in humidity (i.e. “humidity-range”) on virus seasonality.

Our data shows that all the viral agents of URTI show degrees of seasonal variation corroborated by the GLM. Based on similarity in structures and seasonality, there appear to be three groups. 1: Non-enveloped viruses (rhinovirus and adenovirus) which are present throughout the year. 2: Enveloped viruses with winter preponderance (RSV, HMPV, IAV and IBV) and 3: Parainfluenza viruses 1–3, enveloped viruses with a preference for warmer temperatures.

In our study, non-enveloped agents are present year-round with some seasonal variation. Adenovirus is known to be stable at high humidity levels (80%)^19^. Since this is very close to the mean humidity for the study period (81%), this could explain the presence of adenovirus throughout the year. Davis GW showed that adenovirus survival is better at high humidity levels (89%) than at lower humidity (50%)^20^. The peak in March found in our GLM for adenoviruses is different to the December peaks observed for enteric adenoviruses in Japan, though a peak in the winter months is seen when GLM was drawn for 2011, a year when visual peaks for all viruses were present (Supplementary Fig. 1)^21^. Our results show that adenoviruses prefer temperatures around 9 °C. The inverse relationship between adenoviruses and temperature, corroborated by the lower OR, is in keeping with the report from Germany^18^.

The year-round presence of rhinoviruses is similar to other reports^18,22^, but the November peak found using the GLM is later than the September-October peaks found in other research. However, as these two peaks correspond to when children go back to school, it is possible that meteorological factors do not solely account for these peaks.

As rhinovirus is present throughout the year, there appears to be no significant difference in meteorological factors on days rhinovirus is present or absent. This is unlike the negative correlation with temperature and a positive correlation with relative humidity reported from Germany^18^. Whether this reflects the climatic conditions of the UK (being an island) compared to Germany needs further evaluation.

In our study, enveloped viruses with peaks in the winter months, had an association with lower temperatures, dew points and humidity-ranges, and preference for higher humidity. A significant relationship with low temperature, dew point and high relative humidity have been reported for RSV^23,24^, IAV^18,25^ and IBV^25^. RSV and IAV appear to be associated with lower temperatures, dew point, “humidity-range” and high humidity, however, by logistic regression significant association is only seen with lower temperatures and lower “humidity-ranges”. IBV and HMPV which appear to be associated with lower temperatures, dew point, and “humidity-range”, are only associated with lower temperature by logistic regression. This illustrates the complex interaction between different meteorological factors and hence the difficulty in understanding their specific effects on viral prevalence. IBV has a biennial pattern in Edinburgh unlike the bimodal pattern from Okinawa, a subtropical region in Japan^26^. This suggests that local meteorological variation and factors affecting transmission and herd immunity might have a role to play in viral seasonality. Other studies have also reported early spring peaks of HMPV, association with low temperature^27,28^ and raised relative humidity^29^. The biennial pattern for HMPV reported from Germany^30^, Austria^30^ and Sweden^31^, is not seen in our study.

Both RSV and IAV appear when the relative humidity is high (83% to 84%) in Edinburgh. Others have reported variable findings, with some reporting a positive association^18^, and others reporting that enveloped viruses are better transmitted in low humidity^32^. For example, Iha Y *et al*. reported an inverse correlation with relative humidity for influenza A and a direct association with relative humidity for influenza B from Japan^26^. Since relative humidity is significantly associated only with HPIV-3 on logistic regression, the absolute value of humidity may be less important than fluctuation in humidity to the survival of the virus, particularly with viruses predominantly seen in the Autumn and Winter. It is possible that temperature and “humidity-range” could both have a combined impact on viral stability. In laboratory conditions, RSV survives for longer at lower temperatures, with infectivity falling rapidly as temperature increases. It is known that freezing and thawing leads to marked reduction in infectivity of RSV^33^. The preference for a narrow “humidity-range” and the change in relative humidity associated with changes in temperature^34^ could explain why viruses are not stable in frost-free freezers which have to warm up regularly to prevent accumulation of ice in the freezer compartment. This needs further investigation. HPIV 1-3, despite being enveloped RNA viruses, seems to prefer warmer temperatures (8.2 °C-9.4 °C) than the other enveloped RNA viruses. Due to its low incidence over our study period, the seasonality of HPIV-1 and HPIV-2 remain unclear though the GLM suggests it is around October-November. There is a possibility of a biennial seasonality for HPIV-1, similar to that reported in the US^35^, but the trend remains unclear due to the low prevalence in our study. Unlike HPIV-1 and 2, HPIV-3 increases when temperature is highest (9.4 °C) and when relative humidity is lowest (79.9%). HPIV-3 also appears to tolerate large variations in “humidity-range” (29.7%). Following logistic regression, HPIV-3 is associated with lower relative humidity. The seasonal peak for HPIV-3 in May^35^, its stability at lower relative humidity^36^ and higher temperatures^28,36^ have been reported previously. Our results indicate that most viruses prefer stable humidity (i.e. a lower “humidity-range”) through the day. This requires confirmation from further studies.

There are some limitations to this study. There is a possibility that the samples tested may include those who do not live in the catchment area. As the data is anonymised, it is not possible to determine whether the individuals are tourists or residents of the area. However, since all samples tested over the time period were analysed we believe that it is as representative as it can be. And, as the incubation period in viral respiratory infections is relatively short, it is unlikely that a viral infection will have originated abroad unless the patient was infected just prior to travelling to Edinburgh, which should be a relatively small percentage of the overall population examined in this study. Another limitation is that our data only represents those who sought medical care and were then tested. Hence a time lag between the individual becoming infected, becoming symptomatic and subsequently becoming unwell enough to require medical attention is present. We have not corrected our data for incubation period or for duration of illness prior to sampling. We have analysed the proportion of positive results for each virus for each day. Since proportion depends both on the number of positive results and on the total number of samples tested, it does not directly reflect the number of positive cases. We did not analyse coronavirus seasonality as it was not part of the routine testing panel during that period. Since meteorological factors can be interlinked (e.g. dew point and relative humidity), examining the effects of individual meteorological factors on viral seasonality can lead to an incomplete picture^37^. We hence attempted to tease this out by performing logistic regression on variables which showed an association in the univariate analysis. Since dew point was removed to prevent collinearity affecting the results of logistic regression, a new regression could in the future be performed to analyse the impact of dew point by removing temperature. There is a preference for stable “humidity-range” in most enveloped viruses except for HPIV-3. Following a visual inspection of the minimum and maximum for each variable and comparison with virus seasonality patterns for different viruses, there appeared to be a relationship between “humidity-range” and viral seasonality. Hence, we investigated the relationship between “humidity-range” and viral seasonality. It would be interesting to assess if a similar relationship is seen with other ranges such as “temperature-range”, “dew point-range”, etc in the future. Relationship with other variables appears to be virus specific. Therefore, we need models which can investigate multiple variables for each virus. Changes in weather, human behaviour and the immune system can all affect an agent’s seasonality and are all interlinked, as seen in the decrease in vitamin D in temperate climates due to the decrease in daylight hours (shorter/cloudier days). This also makes it difficult to describe the effect of weather on viral seasonality in isolation. Finally, the GLM used in this study has some disadvantages. The GLM used does not account for the lag period between people becoming infected and then seeking medical help, and it also cannot model the multiple peaks seen for rhinoviruses. Furthermore, average data was used to calculate the GLM. This has led to some of the curves not being as smooth as when yearly data is analysed (for e.g. from 2011 a year when peaks were seen for all viruses (Supplementary Fig. 1)). The two steps seen in the curves correspond to the start and end date and probably reflect the lack of samples from January to March in 2009 and the month of December in 2015 as we have analysed data from April 2009 and November 2015. Despite this, the average data over the 6.5 years is very similar to that from 2011. This helps confirm the seasonality for most viruses. There may be differences in individual years as can be seen for adenovirus which had a peak seasonality in December in 2011.

Nonetheless, our findings on temperature, humidity and “humidity-range” could potentially help explain the variation in seasonality between temperate and tropical regions of the world. IAV is known to occur throughout the year in South East Asian countries closer to the equator (warm, humid environment)^38^. The further you are from the equator, winter seasonality and or association with monsoon rains is notable^39,40^. Our findings on “humidity-ranges” need confirmation in a tropical setting. The order of occurrence of different viruses in tropical and temperate regions needs to be investigated in relation to the parameters highlighted. Additionally, whether these peaks in viral activity change depending on the age of the population analysed could also be a topic for future research.

In conclusion, non-enveloped viruses were present throughout the year. Enveloped viruses were more seasonal starting from IAV in January to RSV in December with other viruses in between. RSV, HPIV-3 and IAV and IBV showing the clearest patterns. RSV, IAV and IBV show a clear preference for colder temperature and dew point, as well as consistent humidity. In contrast, HPIV showed a preference for higher temperatures. Additionally, HPIV-3 demonstrated a significant tolerance to low humidity. Hence, meteorological factors like temperature, humidity and “humidity-range” have a significant effect on the incidence of the causative agents of the common cold and changes in meteorological factors could potentially predict the decline of one virus and the emergence of the next.

## Method

### Meteorological data

Following discussion with the Met Office, Edinburgh Gogarbank weather station, which captures data on an hourly basis, was identified as the most representative weather station for the catchment areas examined in this study. We downloaded meteorological data collected by the Edinburgh Gogarbank weather station from the Met Office Weather Observation Website. Hourly air temperature (°C), air pressure (hPa), relative humidity (%) dew point (°C), wind speed and daylight length measurements from 1^st^ April 2009 to the 30^th^ November 2015 were available. Small periods of weather data were missing in October 2011 and June 2015 (reasons unknown). We calculated means and ranges for each variable for each day. We also created weekly and monthly aggregations of the data. As rhinoviruses and HMPV testing only started from the 4^th^ of September 2009, results for these two agents were only available after this date.

### Virological Data

We collated anonymised virological results on all forms of respiratory samples (nose/throat swabs, nasopharyngeal aspirates, bronchoalveolar lavages, endotracheal aspirates, etc) tested at the Department of Laboratory Medicine, Royal Infirmary of Edinburgh. These included all samples from patients attending 8 hospitals around Edinburgh (Astley Ainslie Hospital, Chalmers Centre, Liberton Hospital, Royal Edinburgh Hospital, Royal Hospital for Sick Children, Royal Infirmary of Edinburgh, Royal Victoria Hospital, Western General Hospital), and 126 primary care centres across in Edinburgh and Lothian, tested at the Department of Laboratory Medicine, Royal Infirmary of Edinburgh. We had results from 36,191 individual patients over the study period. Of these patients, 17,936 were male and 18,241 were female (information on gender was not available for 14). The age groups of the cohort are in Table 2. PCR results were available for rhinovirus, adenovirus, IAV, IBV, HPIV 1-3, RSV and HMPV. There were no major modifications to the assay in that time. Rhinovirus and HMPV data was unavailable until screening commenced in September 2009. Between 16^th^-24^th^ February 2011, samples were not tested for rhinovirus due to high work load. Between September 15^th^ and December 9^th^ 2010, IBV was not tested due to technical reasons. We removed repeat positive samples from the same individual collected within 14 days from analysis, resulting in a total of 52060 samples for analysis. We calculated the proportion of positive results (as %) for individual viruses for each day, to help compare results over time. We used daily data for statistical analysis to avoid loss of accuracy from averaging data. To identify seasonal trends, we plotted the proportion of positive results for each day against individual meteorological factors.

### Analysis

Univariate analysis of continuous variables (mean temperature, dew point, relative humidity, and the difference between maximum and minimum humidity in a day; “humidity-range”) were compared between days where one or more samples were positive for a given virus, and days where no samples were positive for that virus using two-sample t-tests. To determine the impact of multiple factors, multivariable logistic regression was conducted using those variables with a univariate p-value of <0.1 in the t-test and models were built using the “Enter (forced entry)” method on SPSS. Due to the likelihood of collinearity affecting results because of the close relationship between dew point and temperature, dew point was removed from the logistic regression.

An analysis of variance (ANOVA) in conjunction with Tukey’s range test were done, to determine if the mean temperature, dew point, pressure, relative humidity, and “humidity-range” at which individual viruses were present were different between viruses. This was performed in conjunction with post-hoc analysis.

Finally, seasonality of individual viruses identified in our study was confirmed with a generalised linear model (GLM) based on temperature following the method of Naumova *et al*.^41^. The GLM was calculated using daily incidence of each virus, averaged over the entire study period.

## Supplementary information

Supplementary Figure 1

## Data Availability

The datasets generated during and/or analysed during the current study are available from the corresponding author on reasonable request.

## References

[1] Jackson GG, Dowling HF, Spiesman IG, Boand AV (1958). Transmission of the common cold to volunteers under controlled conditions. I. The common cold as a clinical entity. AMA Arch Intern Med.

[2] Barrett B (2009). Validation of a short form Wisconsin Upper Respiratory Symptom Survey (WURSS21). Health Qual Life Outcomes.

[3] Zhang SY (2016). Fatal pneumonia cases caused by human adenovirus 55 in immunocompetent adults. Infect Dis (Lond).

[4] Moreno-Perez D, Calvo C, Five Study G (2014). Epidemiological and clinical data of hospitalizations associated with respiratory syncytial virus infection in children under 5 years of age in Spain: FIVE multicenter study. Influenza Other Respir Viruses.

[5] Eccles R (2005). Understanding the symptoms of the common cold and influenza. Lancet Infect Dis.

[6] Ruohola A (2009). Viral etiology of common cold in children, Finland. Emerg Infect Dis.

[7] Jacobs SE, Lamson DM, St George K, Walsh TJ (2013). Human rhinoviruses. Clin Microbiol Rev.

[8] Heikkinen T, Jarvinen A (2003). The common cold. Lancet.

[9] Fisman D (2012). Seasonality of viral infections: mechanisms and unknowns. Clin Microbiol Infect.

[10] Martineau AR (2017). Vitamin D supplementation to prevent acute respiratory tract infections: systematic review and meta-analysis of individual participant data. BMJ.

[11] Lofgren E, Fefferman NH, Naumov YN, Gorski J, Naumova EN (2007). Influenza seasonality: underlying causes and modeling theories. J Virol.

[12] Shaw Stewart PD (2016). Seasonality and selective trends in viral acute respiratory tract infections. Med Hypotheses.

[13] Lowen AC, Mubareka S, Steel J, Palese P (2007). Influenza virus transmission is dependent on relative humidity and temperature. PLoS Pathog.

[14] Polozov IV, Bezrukov L, Gawrisch K, Zimmerberg J (2008). Progressive ordering with decreasing temperature of the phospholipids of influenza virus. Nat Chem Biol.

[15] Pica N, Bouvier NM (2012). Environmental factors affecting the transmission of respiratory viruses. Curr Opin Virol.

[16] Shaman J, Kohn M (2009). Absolute humidity modulates influenza survival, transmission, and seasonality. Proc Natl Acad Sci USA.

[17] Wiemken TL (2017). Impact of Temperature Relative Humidity and Absolute Humidity on the Incidence of Hospitalizations for Lower Respiratory Tract Infections Due to Influenza, Rhinovirus, and Respiratory Syncytial Virus: Results from Community-Acquired Pneumonia Organization (CAPO) International Cohort Study. The University of Louisville Journal of Respiratory Infections.

[18] du Prel JB (2009). Are meteorological parameters associated with acute respiratory tract infections?. Clin Infect Dis.

[19] Miller WS, Artenstein MS (1967). Aerosol stability of three acute respiratory disease viruses. Proc Soc Exp Biol Med.

[20] Davis GW, Griesemer RA, Shadduck JA, Farrell RL (1971). Effect of relative humidity on dynamic aerosols of adenovirus 12. Appl Microbiol.

[21] Dey SK, Hoq I, Okitsu S, Hayakawa S, Ushijima H (2013). Prevalence, seasonality, and peak age of infection of enteric adenoviruses in Japan, 1995-2009. Epidemiol Infect.

[22] Monto AS (2002). The seasonality of rhinovirus infections and its implications for clinical recognition. Clin Ther.

[23] Tang JW, Loh TP (2014). Correlations between climate factors and incidence–a contributor to RSV seasonality. Rev Med Virol.

[24] Yusuf S (2007). The relationship of meteorological conditions to the epidemic activity of respiratory syncytial virus. Epidemiol Infect.

[25] Chan PK (2009). Seasonal influenza activity in Hong Kong and its association with meteorological variations. J Med Virol.

[26] Iha Y (2016). Comparative epidemiology of influenza A and B viral infection in a subtropical region: a 7year surveillance in Okinawa, Japan. BMC Infect Dis.

[27] Sundell N, Andersson LM, Brittain-Long R, Lindh M, Westin J (2016). A four year seasonal survey of the relationship between outdoor climate and epidemiology of viral respiratory tract infections in a temperate climate. J Clin Virol.

[28] Chen ZR (2014). Etiology of acute bronchiolitis and the relationship with meteorological conditions in hospitalized infants in China. J Formos Med Assoc.

[29] Chow WZ (2016). Genetic diversity, seasonality and transmission network of human metapneumovirus: identification of a unique sub-lineage of the fusion and attachment genes. Sci Rep.

[30] Weigl JA (2007). Ten years’ experience with year-round active surveillance of up to 19 respiratory pathogens in children. Eur J Pediatr.

[31] Rafiefard F, Yun Z, Orvell C (2008). Epidemiologic characteristics and seasonal distribution of human metapneumovirus infections in five epidemic seasons in Stockholm, Sweden, 2002-2006. J Med Virol.

[32] Gustin KM (2015). Environmental Conditions Affect Exhalation of H3N2 Seasonal and Variant Influenza Viruses and Respiratory Droplet Transmission in Ferrets. PLoS One.

[33] Hambling MH (1964). Survival of the Respiratory Syncytial Virus during Storage under Various Conditions. Br J Exp Pathol.

[34] Wang Z (2014). Relative humidity and deterioration of concrete under freeze–thaw load. Construction and Building Materials.

[35] Fry AM (2006). Seasonal trends of human parainfluenza viral infections: United States, 1990–2004. Clin Infect Dis.

[36] Elazhary MA, Derbyshire JB (1979). Aerosol stability of bovine parainfluenza type 3 virus. Can J Comp Med.

[37] Lawrence MG (2005). The Relationship between Relative Humidity and the Dewpoint Temperature in Moist Air: A Simple Conversion and Applications. Bull. Am. Meteorol. Soc..

[38] Saha S (2014). Influenza seasonality and vaccination timing in tropical and subtropical areas of southern and south-eastern Asia. Bull World Health Organ.

[39] Chadha MS (2015). Dynamics of influenza seasonality at sub-regional levels in India and implications for vaccination timing. PLoS One.

[40] Hirve S (2016). Influenza Seasonality in the Tropics and Subtropics - When to Vaccinate?. PLoS One.

[41] Naumova EN (2007). Seasonality in six enterically transmitted diseases and ambient temperature. Epidemiol Infect.

