# Supplementary material for: Association between viral seasonality and meteorological factors
Source: Sci Rep. 2019 Jan 30;9:929. doi: 10.1038/s41598-018-37481-y (PMC6353886; doi:10.1038/s41598-018-37481-y)

Association between viral seasonality and meteorological factors

Authors:

Rory Henry Macgregor Price[1,2], Catriona Graham[3], Sandeep Ramalingam[1, 2*],

1. Department of Laboratory Medicine, Royal Infirmary of Edinburgh, UK
2. Division of Infection and Pathway Medicine, Division of Infection and Pathway Medicine, University of Edinburgh, UK
3. Wellcome Trust Clinical Research Facility, University of Edinburgh, UK

***Correspondence:**

Dr. Sandeep Ramalingam

Consultant Virologist

Department of Laboratory Medicine

Royal Infirmary of Edinburgh

51 Little France Crescent

Edinburgh

EH16 4SA

Email: Sandeep.Ramalingam@nhslothian.scot.nhs.uk

Supplementary Figure 1: Generalised Linear Models for 2011.

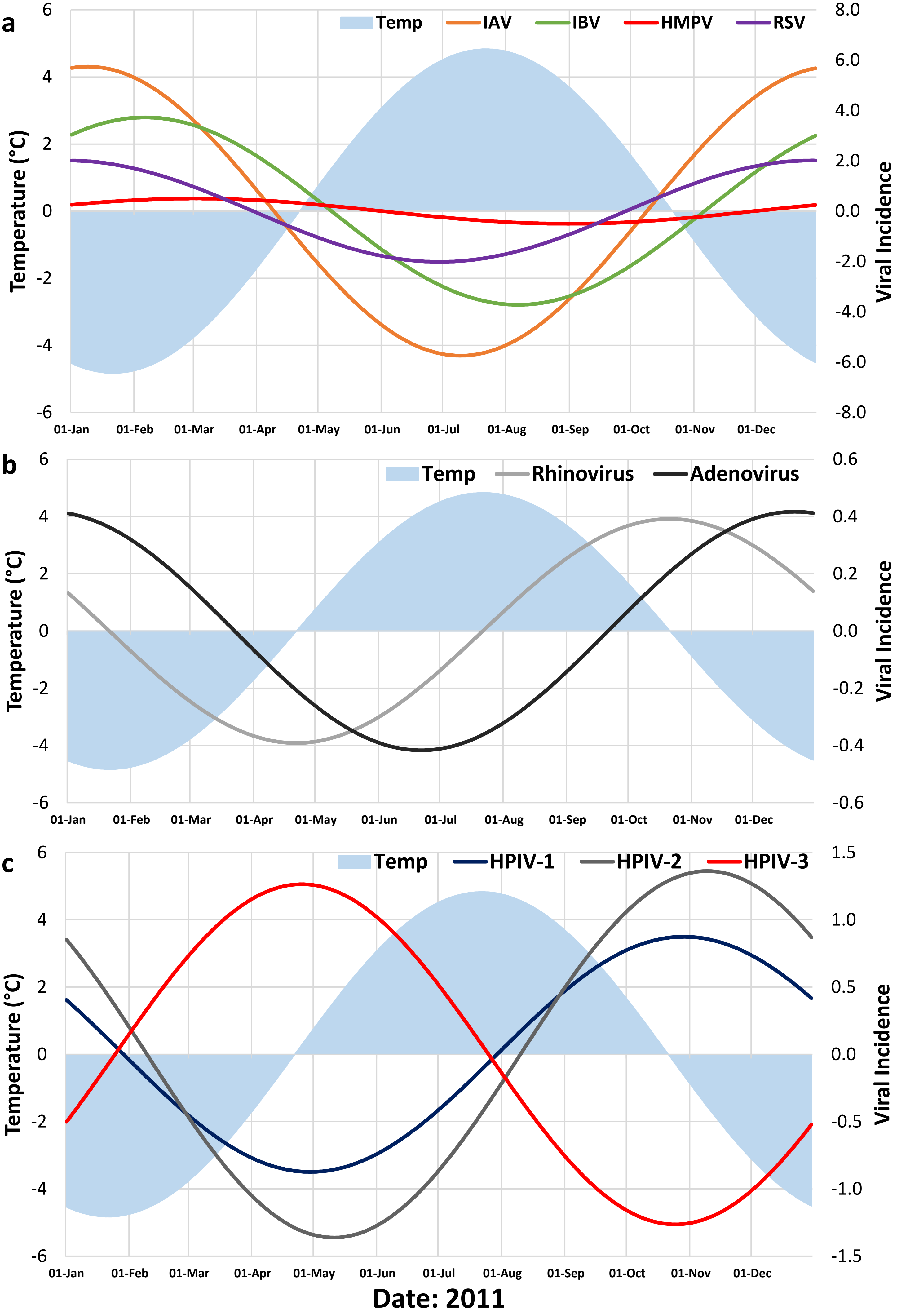

Supplement: Supplementary file 1 — Supplementary Figure 1 [file 41598_2018_37481_MOESM1_ESM.pdf]
